# Fluoride Alters Klk4 Expression in Maturation Ameloblasts through Androgen and Progesterone Receptor Signaling

**DOI:** 10.3389/fphys.2017.00925

**Published:** 2017-11-14

**Authors:** Michael H. Le, Yukiko Nakano, Dawud Abduweli Uyghurturk, Li Zhu, Pamela K. Den Besten

**Affiliations:** ^1^Department of Orofacial Sciences, School of Dentistry, University of California, San Francisco, San Francisco, CA, United States; ^2^Center for Children's Oral Health Research, School of Dentistry, University of California, San Francisco, San Francisco, CA, United States

**Keywords:** ameloblasts, enamel, fluoride, KLK4, AR, PR, HSP90, TGF-β

## Abstract

Fluorosed maturation stage enamel is hypomineralized in part due to a delay in the removal of matrix proteins to inhibit final crystal growth. The delay in protein removal is likely related to reduced expression of kallikrein-related peptidase 4 (KLK4), resulting in a reduced matrix proteinase activity that found in fluorosed enamel. *Klk4* transcription is known to be regulated in other cell types by androgen receptor (AR) and progesterone receptors (PR). In this study, we determined the possible role of fluoride in down-regulation of KLK4 expression through changes in AR and PR. Immunohistochemical localization showed that both AR and PR nuclear translocation was suppressed in fluoride exposed mice. However, when AR signaling was silenced in mouse ameloblast-lineage cells (ALCs), expression of both *Pgr* and *Klk4* were increased. Similar to the effect from AR silencing, fluoride also upregulated *Pgr* in ALCs, but downregulated *Klk4*. This finding suggests that though suppression of AR transactivation by fluoride increases *Prg* expression, inhibition of PR transactivation by fluoride has a much greater effect, ultimately resulting in downregulation of *Klk4* expression. These findings indicate that in ameloblasts, PR has a dominant role in regulating *Klk4* expression. We found that when AR was retained in the cytoplasm in the presence of fluoride, that co-localized with heat shock protein 90 (HSP90), a well-known chaperone for steroid hormone receptors. HSP90 also known to regulate TGF-β signaling. Consistent with the effect of fluoride on AR and HSP90, we found evidence of reduced TGF-β signaling activity in fluorosed ameloblasts as reduced immunolocalization of TGFB1 and TGFBR-2 and a significant increase in Cyclin D1 mRNA expression, which also possibly contributes to the reduced AR signaling activity. *In vitro*, when serum was removed from the media, aluminum was required for fluoride to inhibit the dissociation of HSP90 from AR. In conclusion, fluoride related downregulation of *Klk4* is associated with reduced nuclear translocation of AR and PR, and also reduced TGF-β signaling activity, all of which are regulated by HSP90. We suggest that a common mechanism by which fluoride affects AR, PR, and TGF-β signaling is through inhibiting ATP-dependent conformational cycling of HSP90.

## Introduction

Enamel formed in the presence of high levels of fluoride has a delayed removal of matrix proteins (Den Besten, 1986; DenBesten and Thariani, 1992), most likely due to reduced proteolytic activity in the maturation stage fluorosed enamel (DenBesten et al., 2002). Reduced proteolytic activity in maturation stage fluorosed enamel is consistent with reduced expression of kallikrein-related peptidase 4 (KLK4) (Suzuki et al., 2014), the serine proteinase that is responsible for the final hydrolysis of matrix proteins, which allows enamel hydroxyapatite crystals to reach their full thickness.

KLK4 expression in cancer cell lines, has been shown to be regulated by activation of both androgen receptor (AR) and progesterone receptor (PR) (Lai et al., 2009). AR and PR are members of the nuclear receptor superfamily of transcription factors, residing predominantly in the cytoplasm. Binding of the ligand (i.e., androgenic hormones) promotes nuclear translocation and transactivation for transcription of the target genes (Azad et al., 2015). Jedeon and co-workers have reported that the AR is present in ameloblasts and when activated, regulates KLK4 expression (Jedeon et al., 2016).

Suzuki et al. found that fluoride related downregulation of *Klk4* expression is associated with reduced TGF-β signaling. AR signaling also involves cross-talk with the TGF-β signaling pathway (Gerdes et al., 1998; Bruckheimer and Kyprianou, 2001; Chipuk et al., 2002; Kang et al., 2002; Pratt et al., 2004; Yang et al., 2014), suggesting that possibility that fluoride related effects on TGF-β signaling are also related to effects of the fluoride on AR activation.

To explore the intracellular mechanisms that mediate effects of fluoride on *Klk4* transcription, we used both *in vivo* and *in vitro* experimental models to examine the effects of fluoride on AR, PR, and KLK4 in fluorosed maturation ameloblasts. We found that fluoride related downregulation of *Klk4* was associated with reduced nuclear translocation of AR and PR, and also reduced TGF-β signaling activity, all of which are regulated by heat shock protein 90 (HSP90). *In vitro*, we found that fluoride together with aluminum induced a cytoplasmic retention of AR, suggesting the possibility that fluoride requires aluminum to inhibit the chaperone function of HSP90 to regulate AR nuclear translocation.

## Materials and methods

### Animals

All animal procedures were carried out with approval by the University of California-San Francisco Institutional Animal Care and Use Committees. The experiments reported herein were conducted in compliance with the Animal Welfare Act and in accordance with the principles set forth in the National Research Council's *Guide for the Care and Use of Laboratory Animals*.

To determine whether the reduced *Klk4* expression in the presence of fluoride resulted in decreased KLK4 activity in the enamel matrix, 3-week-old female Wistar rats (Jackson Laboratory, Sacramento, CA) were divided into two groups, with the groups given either deionized drinking water or deionized drinking water supplemented with 100 ppm (5.3 mM) fluoride as sodium fluoride (Sigma-Aldrich, St. Louis, MO) *ad libitium* for 4 weeks. After 4 weeks, the rats were euthanized, and the mandibular incisors were dissected to allow access to separately dissected the enamel matrix and enamel organ for KLK4 activity assays, and quantitative real-time polymerase chain reaction (qPCR) of the relative expression of *Klk4* mRNA.

Three-week-old C57BL/6J female mice (Jackson Laboratory) were divided into two groups, with the groups given either deionized drinking water or deionized drinking water supplemented with 50 ppm (2.6 mM) fluoride as sodium fluoride (Sigma-Aldrich) *ad libitium* for 4 weeks. After 4 weeks, the mice were euthanized, and mandibles were obtained for morphology, immunohistochemical, and qPCR analyses.

### KLK4 activity assay

Rat mandibular incisors were removed from the alveolar bone and enamel matrix was dissected from the underlying dentin surface at the early-maturation stage. This includes enamel matrix underlying the distal root of the first molar and continuing until the enamel was too hard to dissect (Stahl et al., 2015). Extracts were homogenized in 100 μl of 5% TCA, and incubated at room temperature for 30 min. After centrifugation at 10,000 × g for 10 min, the supernatants were removed and pellets were re-suspended in 200 μl of ammonium bicarbonate. The total protein concentration of each sample was measured by Bradford assay. KLK4 activity was analyzed by using Boc-Val-Pro-Arg-AMC fluorogenic peptide substrate (R&D Systems Inc., Minneapolis, MN). Each reaction in 96-well black plates contained 150 μl of protein extracts, 5 μl of peptide and 40 μl of 5x reaction buffer (250 mM Tris–HCl, 250 mM NaCl, 50 mM CaCl). The fluorescence signal was detected using a spectrometer at 37°C (excitation at 380 nm and emission at 460 nm), and measured every 20 min for 180 min. For each sample, the fluorescence data was normalized to the protein concentration of a given sample. For each sample, we used R software environment with *drc* package (Ritz and Streibig, 2005; Team, 2014) to generate an averaged 4-parametric regression curve of the treatment groups. Significance of differences at 60, 120, and 180 min were determined by independent Student's *t*-test.

### qPCR analysis

Total RNA was isolated from (rat and mouse) mandibular maturation-stage incisor enamel organs that were dissected according to landmarks described previously (Stahl et al., 2015), and also from ameloblast lineage cells (ALCs; detail in Cell Culture) using RNeasy Mini kit (Qiagen, Germantown, MD). Conversion of mRNA to cDNA was obtained by reverse transcription of the mRNA using Superscript III First-Strand Synthesis Supermix for qRT-PCR (Life Technologies, Carlsbad, CA).

Expression of mRNAs was examined by qPCR with FastStart Universal SYBR Green Master Kit (Roche Diagnostics, Indianapolis, IN) using primer sets for *Klk4, Ccnd1, Ar, Tgfbr2*, and *Tgfb1* (Elim Biopharmaceuticals, Hayward, CA). *Mrpl19* was used as a reference gene for enamel organ samples and *Eef1a1* for ALCs. Primer sequences are listed in Table 1. The relative expression level of target genes was analyzed by the ΔΔCt method (Livak and Schmittgen, 2001). Expression of each gene was calculated as a relative expression level (fold change) compared with WT (mice samples) or untreated controls (cell culture). Significance of differences was determined using ΔCt values by the two-tailed multiple *t*-test with Benjamini & Hochberg correction following ANOVA (Benjamini and Hochberg, 1995).

**Table 1:** Mouse specific primers for qPCR.

**Symbol**	**Accession**	**Region**	
*Ar*	NM_013476	2506–2596	5′-CCCGTCCCAATTGTGTGAAA−3′ 3′-TCCCTGGTACTGTCCAAACG−5′
*Ccnd1*	NM_007631	602–724	5′-TTGTGCATCTACACTGACAAC−3′ 3′-GAAGTGTTCGATGAAATCGT−5′
*Eef1a1*	NM_010106	144–303	5′-CAACATCGTCGTAATCGGACA−3′ 3′-GTCTAAGACCCAGGCGTACTT−5′
*Hsp90*	NM_010480	1532–1647	5′-TGTTGCGGTACTACACATCTGC−3′ 3′-GTCCTTGGTCTCACCTGTGATA−5′
*Klk4*	NM_019928	488–617	5′-GAGATACCTGCCTAGTCTCTGG−3′ 3′-AGGTGGTACACAGGGTCATAC−5′
*Mrpl19*	NM_026490	94–249	5′-ACGGCTTGCTGCCTTCGCAT−3′ 3′-AGGAACCTTCTCTCGTCTTCCGGG−5′
*Pgr*	NM_008829	1001–1122	5′-CTCCGGGACCGAACAGAGT−3′ 3′-ACAACAACCCTTTGGTAGCAG−5′
*Tgfbr2*	NM_029575	654–839	5′-GGCTTCACTCTGGAAGATGC−3′ 3′-GCTGACACCCGTCACTTGGA−5′

### Immunohistochemistry

Mouse mandibles were fixed in 4% paraformaldehyde in 0.06 M sodium cacodylate buffer (pH 7.3) at 4°C for 24 h. After decalcification in 8% EDTA (pH 7.3), samples were processed for routine paraffin embedding and sagittally sectioned. The sections were incubated with 10% swine and 5% goat sera followed by incubation with rabbit anti-human AR (1:75; Novus Biologicals, Littleton, CO, NB100-91658), rabbit anti-mouse TGFBR2 (1:100; Santa Cruz Biotech, Santa Cruz, CA, sc-1700), rabbit anti-human TGFB1 (1:50; Abcam PLC, Cambridge, MA, ab92486), and rabbit anti-mouse PR (Santa Cruz Biotech, sc-166170) antibodies respectively overnight at room temperature. A biotinylated swine anti-rabbit IgG F(ab')2 fraction (Dako, Carpinteria, CA) was used as the secondary antibody for 1 h at room temperature incubation. Following incubation with alkaline phosphatase conjugated streptavidin (Vector Laboratories Inc., Burlingame, CA) for 30 min, immunoreactivity was visualized using a Vector® Red kit (Vector Laboratories) resulting in pink/red color for positive staining. Counter-staining was performed with methyl green (Dako). Negative control was done with normal rabbit sera.

### Cell culture

Mouse ameloblast-lineage cells (ALCs, a kind gift from Dr. Toshihiro Sugiyama, Akita University, Japan and Dr. John Bartlett, Forsyth Institute, Boston, Massachusetts) (Nakata et al., 2003) were cultured in DMEM (UCSF Cell Culture Facility, San Francisco, CA) supplemented with 10% Fetal Bovine Serum (FBS) (Gemini Bio-Products, West Sacramento, CA) and 1% penicillin-streptomycin. ALCs were seeded into 6-well plates (2.0 × 10^5^ cells/cm^2^) or 8-well chamber slides (1.5 × 10^5^ cells/cm^2^). After 24 h, the cells were treated with fluoride (0 or 1 mM) as sodium fluoride (Sigma-Aldrich), followed by total RNA extraction. Other cells, initially cultured in the medium with FBS for 24 h, were exposed to 2% TCM® serum replacement (MP Biomedicals, Santa Ana, CA) for 24 h. After 24 h, cells were transfected with antisense oligonucleotides targeting AR, LNA™ GapmeRs (Exiqon Incorporated, Woburn, MA) to silence *Ar* mRNA, using Lipofectamine 2000 Transfection Reagent (ThermoFisher Scientific, Waltham, MA). Cells were then harvested for total RNA and extracts were analyzed for *Klk4, Ar, Pgr, Tgfb1, Tgfbr2*, and *Ccnd1* expression. For immunofluorescent staining, some cells were treated with fluoride as described above. Other cells were initially cultured in FBS containing medium for 24 h. After culturing in the medium with 2% TCM® serum replacement for another 24 h, cells were treated with 0 or 1 μM dihydrotestosterone/DHT (Cerilliant Corporation, Round Rock, TX). Some of the DHT treated cells were further exposed to aluminum (0, 10, or 100 μM) as aluminum chloride (Sigma-Aldrich) and/or fluoride (0 or 1 mM) as sodium fluoride (Sigma-Aldrich). Aluminum concentration was equivalent to the range of those in calf serum (Tomza-Marciniak et al., 2011)

### Immunofluorescent staining

Cells grown in 8-well chamber slides were fixed in 4% paraformaldehyde (Sigma-Aldrich) for 10 min at room temperature and washed with PBS. They were then permeablized with 0.25% Triton-X 100 and incubated with 10% swine and 5% goat sera to block non-specific binding. Cells were then simultaneously incubated with AR (Novus Biologicals) and HSP90 (Santa Cruz Biotech) antibodies that were fluorescently tagged with Zenon Rabbit IgG Labeling Kit (Molecular Probes, Eugene, OR) per manufacturer's instructions. AR antibody was labeled with Alexa Fluor 594 and HSP90 antibody was labeled with Alex Fluor 488. After antibody incubation, cells were washed with PBS, then counterstained with DAPI and mounted with mounting medium.

## Results

### Fluoride suppresses KLK4 activity *in vivo*

**Figure 1:**
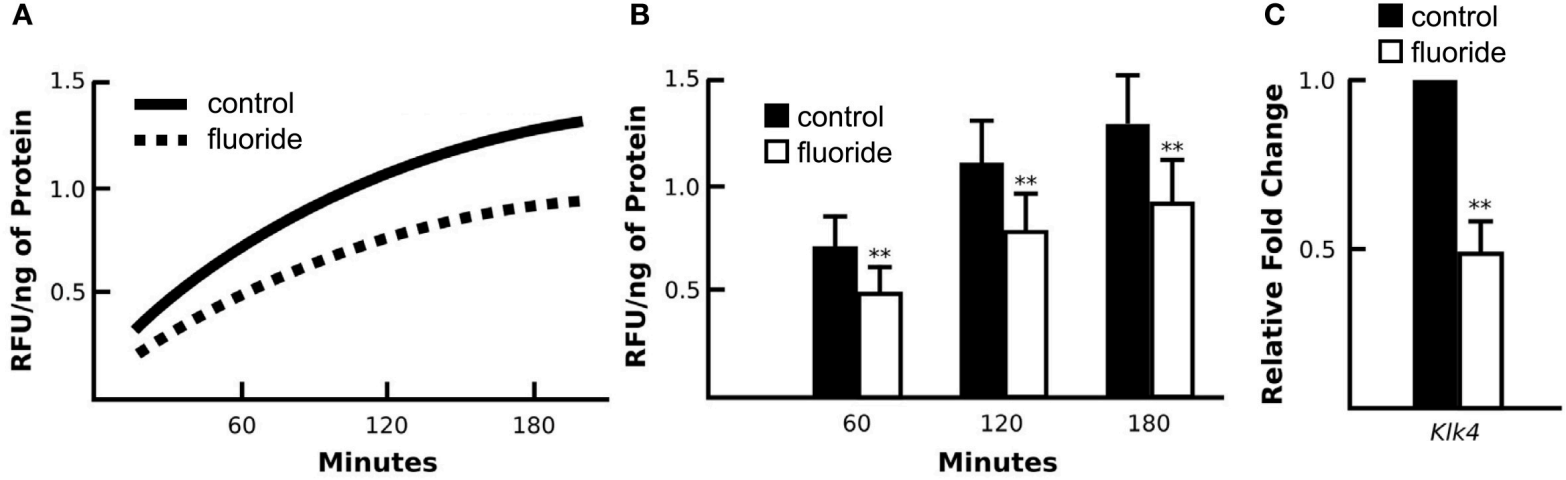
Enamel fluorosis associates with reduced *in vivo* KLK4 activity and *Klk4* expression. **(A)** Averaged profile model of KLK4 activity in early maturation enamel matrix protein extracts (line graph). **(B)** At three time points of measurement (60, 120, and 180 min after the start of incubation), fluorosed enamel showed significantly lower KLK4 activity (bar graph). ^**^p < 0.01. **(C**) Relative fold change of *Klk4* expression from maturation-stage enamel organs harvested from control and fluorosed rats shows reduced *Klk4* expression in fluoride exposed ameloblasts. Fold change was calculated relative to the baseline expression of the gene in control mice. Genes were normalized to the expression of *Mrpl19* (mouse ribosomal protein L19). ^**^p < 0.01.

To determine the ultimate effect of fluoride on KLK4 synthesis, we assessed synthesis of KLK4 in fluorosed rat enamel, by comparing KLK4 proteinase activity in the enamel matrix of rats given either 0 or 100 ppm fluoride in drinking water. Enamel matrix protein extracts of rats given 100 ppm F in drinking water had reduced matrix KLK4 proteolytic activity when compared with control rats (Figure 1A), and significance of the reduction was confirmed at three different time points of incubation (Figure 1B). Consistent with the reduced proteolytic activity in the enamel matrix, *Klk4* mRNA expression was significantly reduced (Figure 1C). Similar to rats, fluorosed mice (given 50 ppm F in drinking water) also had reduced *Klk4* expression (data not shown). To further investigate the effect of fluoride on molecular profiles in maturation ameloblasts, we therefore used the mouse model.

### Nuclear translocation of AR is suppressed in fluorosed maturation ameloblasts *in vivo* and *in vitro*

**Figure 2:**
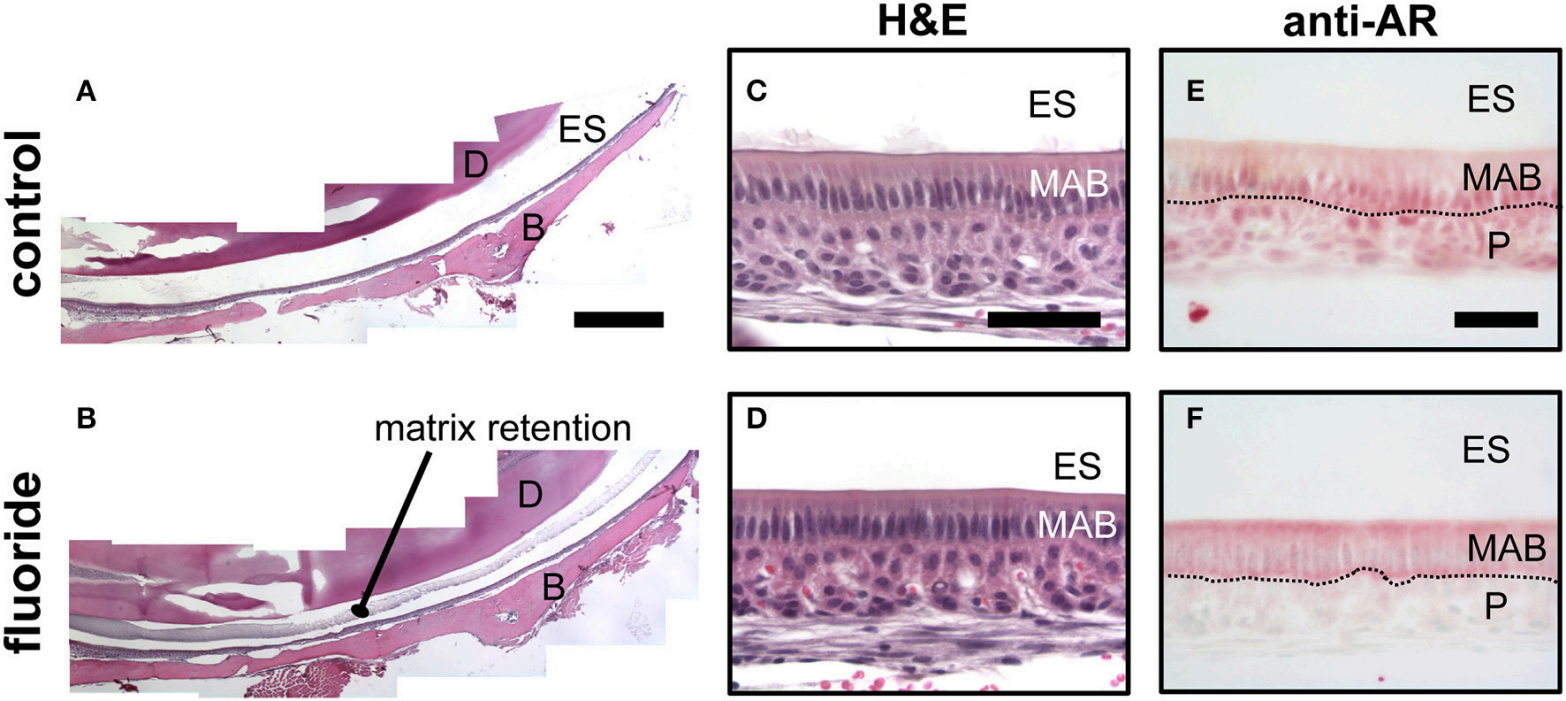
Fluoride related change in the enamel matrix correlates with reduced AR nuclear translocation. **(A–B)** Hematoxylin and eosin-stained mandibular incisors from control **(A)** and fluorosed **(B)** mice. Retained enamel matrix protein is seen in the enamel space of fluorosed but not control mouse enamel. Composite scale bar 1 mm. ES; enamel space, D; dentin, B; bone **(C–D)** Hematoxylin and eosin-stained maturation ameloblasts from control **(C)** and fluorosed **(D)** mice. Bar; 100 um **(E–F)** Maturation-stage ameloblasts immunostained for AR. Control mice have positive AR immunostaining (red color) in the nucleus of maturation ameloblasts (MAB) and papillary layer cells (P). **(E)**. In fluorosed mice, AR immunostaining is mainly seen in the cytoplasm of ameloblasts and absent on the nuclei. Papillary layer cells are negative for AR immunostaining **(F)**. ES; enamel space, Bar; 50 μm.

**Figure 3:**
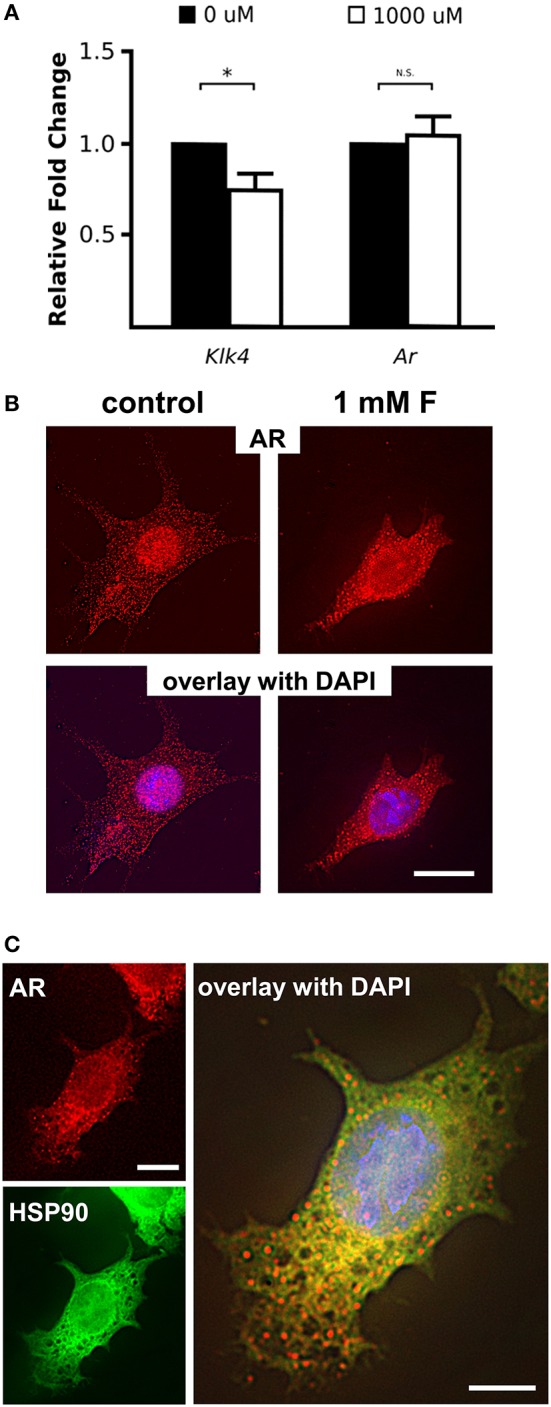
Fluoride reduces *Klk4* expression without altering *Ar* expression. **(A)** Relative fold change of *Klk4* and *Ar* from ALCs exposed to 0 or 1,000 μM fluoride showed significant down regulation of *Klk4* but not *Ar*. Expression of target genes were normalized to the expression of *Mrpl19*. ^*^p < 0.05. **(B)** Immunofluorescent staining of AR (red) in ALC showed a relative reduction in AR nuclear translocation in cells exposed to 1 mM fluoride. Scale bar 25 μm. **(C)** Immunofluorescent staining of AR (in red) and HSP90 (in green) in ALC showed that when AR translocation is blocked by 1 mM fluoride, AR and HSP90 are primarily co-localized in the cytoplasm (in yellow). DAPI (in blue), Scale bar 25 μm.

Mice given high levels of fluoride in drinking water had retained enamel matrix (Figures 2A,B), with no obvious morphological alteration in ameloblasts (Figures 2C,D). To investigate the association of AR with fluorosis, we examined the presence and localization pattern of AR in maturation stage ameloblasts. In control mice, most of the AR immunostaining was in the ameloblast nucleus (Figure 2E), whereas in mice ingesting fluoride, immunostaining showed AR mostly remained in the cytoplasm (Figure 2F). Similarly, *in vitro*, we found that ALCs grown in serum containing medium with 1 mM fluoride had significantly less *Klk4* expression as compared to ALCs grown in medium without fluoride (Figure 3A). Under this condition, *Ar* expression (Figure 3A) and cell proliferation (data not shown) were not changed, but visibly less AR localized in the nuclei (Figure 3B). These *in vivo* and *in vitro* data together show that nuclear translocation of AR is inhibited by fluoride.

### HSP90 co-localizes with cytoplasmic AR in fluorosed ameloblasts

Nuclear translocation of steroid hormone receptors is well-known to be regulated by the heat shock proteins (HSPs) (Azad et al., 2015). HSP90 plays a major role in the activation process of the steroid hormone receptors by interacting with the unliganded steroid hormone receptors to open the steroid-binding cleft to allow access by a steroid (Pratt et al., 2004). Once the ligand binds to the steroid hormone receptors, HSP90 disassociates from the receptors, allowing the nuclear translocation of the steroid receptors to function as a transcription factor (Azad et al., 2015). To determine if the dissociation of HSP90 from AR is altered by fluoride, we co-immunostained AR and HSP90, and observed co-localization of HSP90 and AR remained in the cytoplasm under the influence of fluoride. We found that HSP90 and AR co-localized in the cytoplasm particularly in the perinuclear region (Figure 3C). In the presence of fluoride, mRNA transcription and protein synthesis of HSP90 were unchanged (data not shown).

### Decreased AR activation in fluorosed ameloblasts is related to the TGF-β1- cyclin D1 pathway

Previously, Suzuki et al. reported that fluoride treated ALCs had decreased mRNA expression of *Tgf*β*1* and *Klk4* (Suzuki et al., 2014). In normal and oncogenic prostate cells, AR signaling is known to cross-talk with the TGF-β signaling pathway (Gerdes et al., 1998; Bruckheimer and Kyprianou, 2001; Chipuk et al., 2002; Kang et al., 2002; Pratt et al., 2004; Yang et al., 2014). Furthermore, activation of TGF-β signaling is also regulated by HSP90, and inhibition of HSP90 induces degradation of TGF-β receptors (Wrighton et al., 2008).

**Figure 4:**
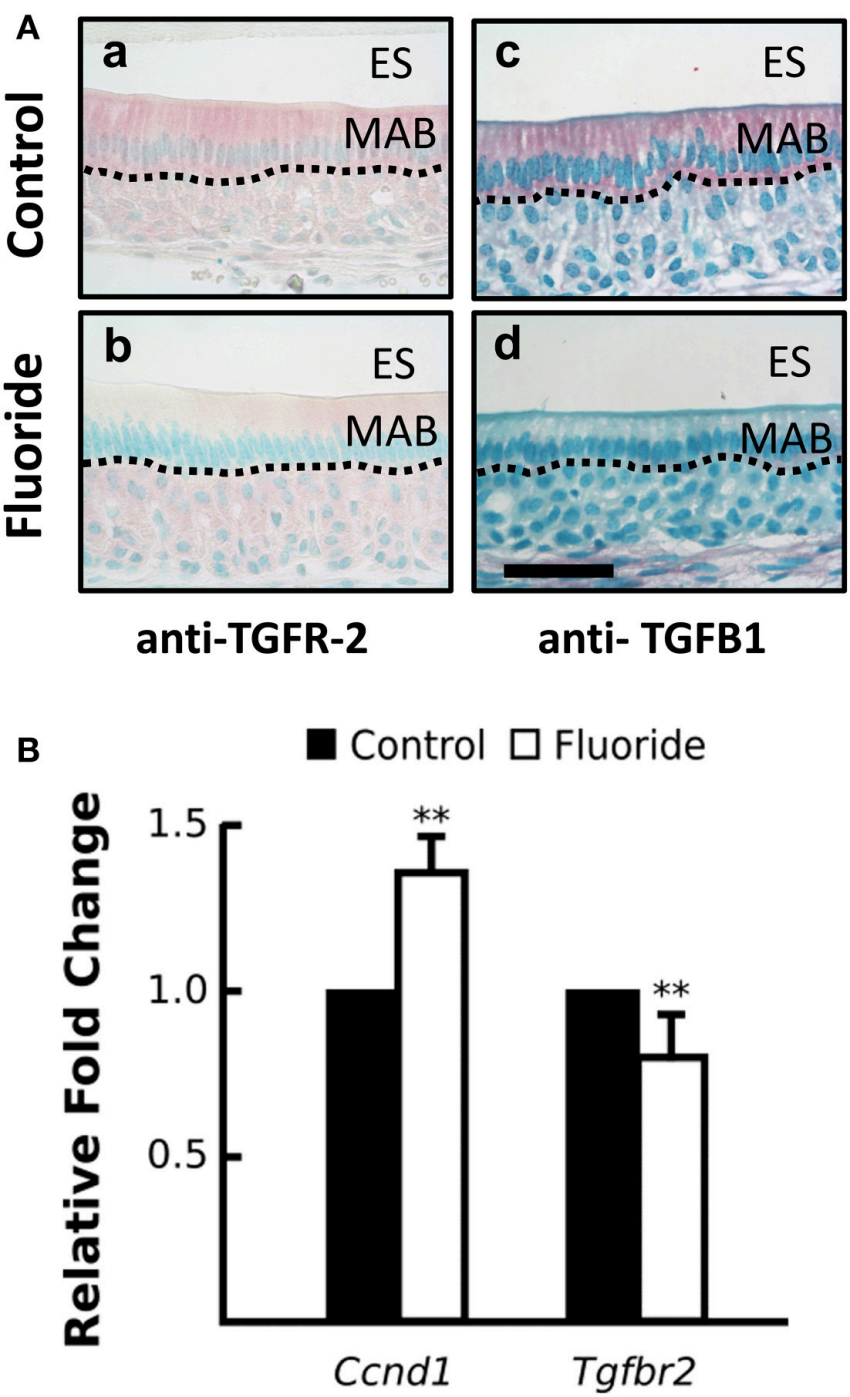
Fluoride altered expression CCND1 and TGFR-2, regulators of androgen receptor activation. **(A)** Images of red TGFR-2 (a–c) and TGFβ1 (b–d) immunostaining show fluoride-exposed maturation ameloblasts have decreased amounts of TGFR-2 (b) and TGFβ1 (d) protein compared to controls (a and c). Scale bar 50 um, ES; enamel space, MAB; maturation ameloblast **(B)** Relative fold change of *Ccnd1* was increased, whereas *Tgfbr2* expression was decreased in maturation-stage ameloblasts harvested from fluorosed as compared to control mice. Genes were normalized to the expression of *Mrpl19*. ^**^p < 0.01

To examine TGF-β signaling in ameloblasts, when fluoride inhibits AR activation *in vivo*, we immunostained maturation ameloblasts from fluoride treated and control mice for TGFB1 and TGF-β1 receptor type II (TGFR-2). In fluorosed ameloblasts, immunostaining for both TGFB1 and TGFR-2 was reduced (Figure 4A). *Tgf*β*r2* transcription was also significantly reduced in fluorosed ameloblasts (Figure 4B), indicating a reduced TGF-β signaling activity. Cyclin D1 (CCND1) is a protein commonly known for regulating cell cycle dynamics, and expression of *Ccnd1* is known to be negatively regulated by TGF-β1 signaling in intestinal epithelial cells (Ko et al., 1995). Moreover, CCND1 is known to further negatively regulate the AR function, not only by directly binding to AR to inhibit transactivation of AR, but also by attenuating AR-induced upregulation of *Klk4* in prostate cancer (Knudsen et al., 1999; Comstock et al., 2011). Supporting our findings of reduced TGF-β signaling and inhibited AR activation in maturation ameloblasts, *Ccnd1 e*xpression in fluorosed ameloblast was significantly increased (Figure 4B). These results suggest that the previously described effects of fluoride on TGF-β signaling, as related to decreased *Klk4* expression, are also possibly mediated by the effects of fluoride on HSP90.

### Suppression of AR expression results in increased expression of PR and Klk4

**Figure 5:**
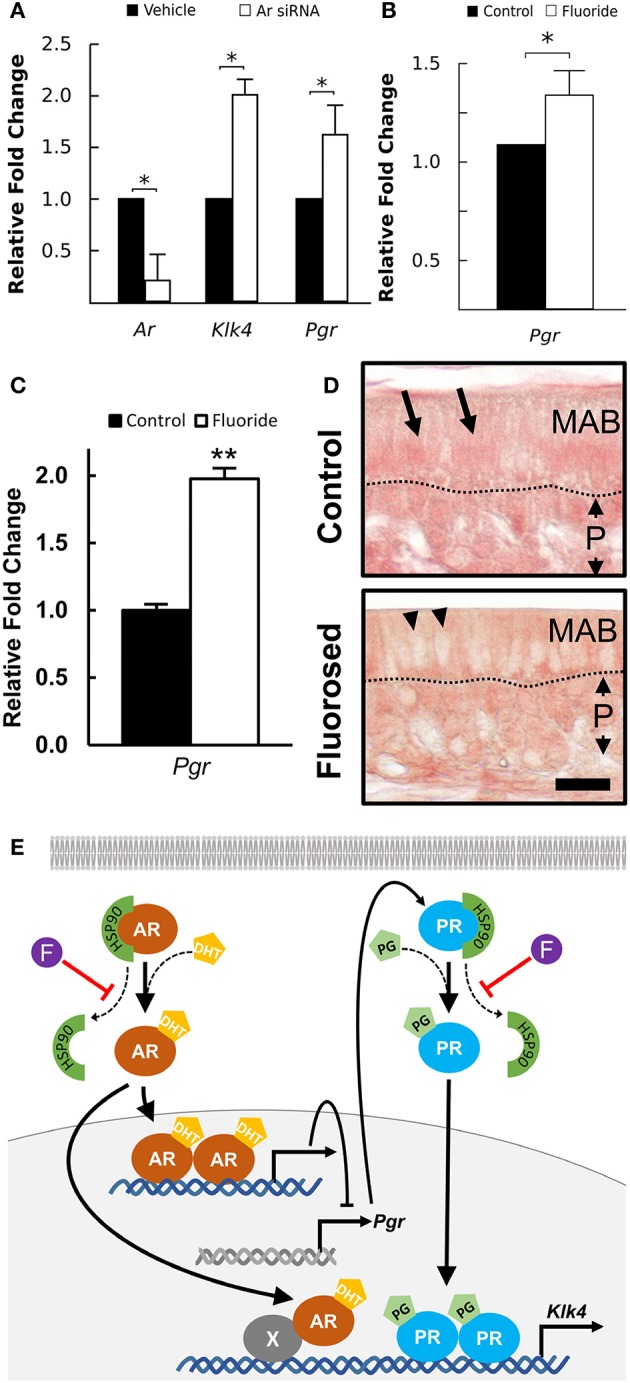
*Klk4* expression is dominantly regulated by PR. **(A)** Relative fold change of *Ar, Klk4*, and *Pgr* mRNA expression of ALCs exposed to antisense LNA™ oligonucleotides targeting AR or a vehicle control. The fold change was calculated relative to the baseline expression in control mice. Expression of the target genes is normalized to the expression of *EeF1a1*. ^*^p < 0.05. **(B)** Relative fold change of *Pgr* from ALCs exposed to 0 or 1 mM fluoride. Expression of target genes were normalized to the expression of *EeFla1*. ^*^p < 0.05, **(C)** Relative fold change of *Pgr* in ameloblast from fluoride exposed mice as compared to controls shows a similar upregulation of *Pgr* expression *in vivo* as compared to *in vitro*. ^**^p < 0.01, **(D)** Immunostaining of PR on maturation-stage ameloblasts. In control mice, intense immunostaining (in red) is seen in the nucleus of ameloblast (MAB, arrows). In fluorosed mice, immunostaining is mainly seen in the cytoplasm of ameloblasts and is absent from the nucleus (arrow heads). P; papillary layer, Scale bar 25 μm. **(E)** A proposed pathway of AR regulation of *Klk4* transcription: AR indirectly interacts with *Klk4* gene via co-regulator (X), to regulate *Klk4* transcription. AR also negatively regulates *Pgr* expression, and when AR is reduced, *Pgr* expression increases. Nuclear translocation of AR and PR is chaperoned by HSP90. Without fluoride, increased *Pgr* results in an upregulated *Klk4* transcription. With fluoride present, nuclear translocation of PR is inhibited, resulting in a final down regulation of *Klk4* transcription.

AR is known to regulate *Klk4* expression, but it does not directly interact with the *Klk4* gene (Lai et al., 2009). PR induces *Klk4* expression in breast cancer cells through direct binding to the *Klk4* promoter (Lai et al., 2009). AR suppresses expression of PR-regulated genes in triple negative breast cancer (Tsang et al., 2014; Karamouzis et al., 2016), suggesting negative regulation of AR on *Pgr* expression. To understand how AR regulates *Klk4* transcription in ameloblasts as well as the potential involvement of PR, we silenced *Ar* mRNA in ALCs and examined the expression of *Klk4* and *Pgr*. The antisense oligonucleotides for *Ar* significantly reduced *Ar* mRNA expression in ALCs, but significantly upregulated both *Klk4* and *Pgr* expression (Figure 5A). This suggests a negative regulation of *Pgr* by AR, while PR directly upregulates *Klk4* expression.

### Fluoride increases Pgr expression, but reduces PR nuclear translocation

To further examine the effect of fluoride induced suppression of AR activation on *Pgr* transcription, we examined *Pgr* expression in ameloblasts of fluorosed mice and in ALC culture. In both *in vitro* (Figure 5B), and *in vivo* (Figure 5C), fluoride exposure resulted in significantly increased *Pgr* expression. As PR nuclear translocation is also chaperoned by HSP90 (Dao-Phan et al., 1997), we examined the change of intracellular localization of PR in fluorosed maturation ameloblasts. Similar to the effects of fluoride on AR, nuclear translocation of PR was reduced in fluorosed ameloblasts as compared to controls (Figure 5D). Therefore, though fluoride increased *Pgr* expression, it inhibited nuclear translocation of both PR and AR, resulting in reduced expression of *Klk4* (see diagram shown in Figure 5E).

### The inhibitory effect of fluoride on AR translocation requires aluminum

**Figure 6:**
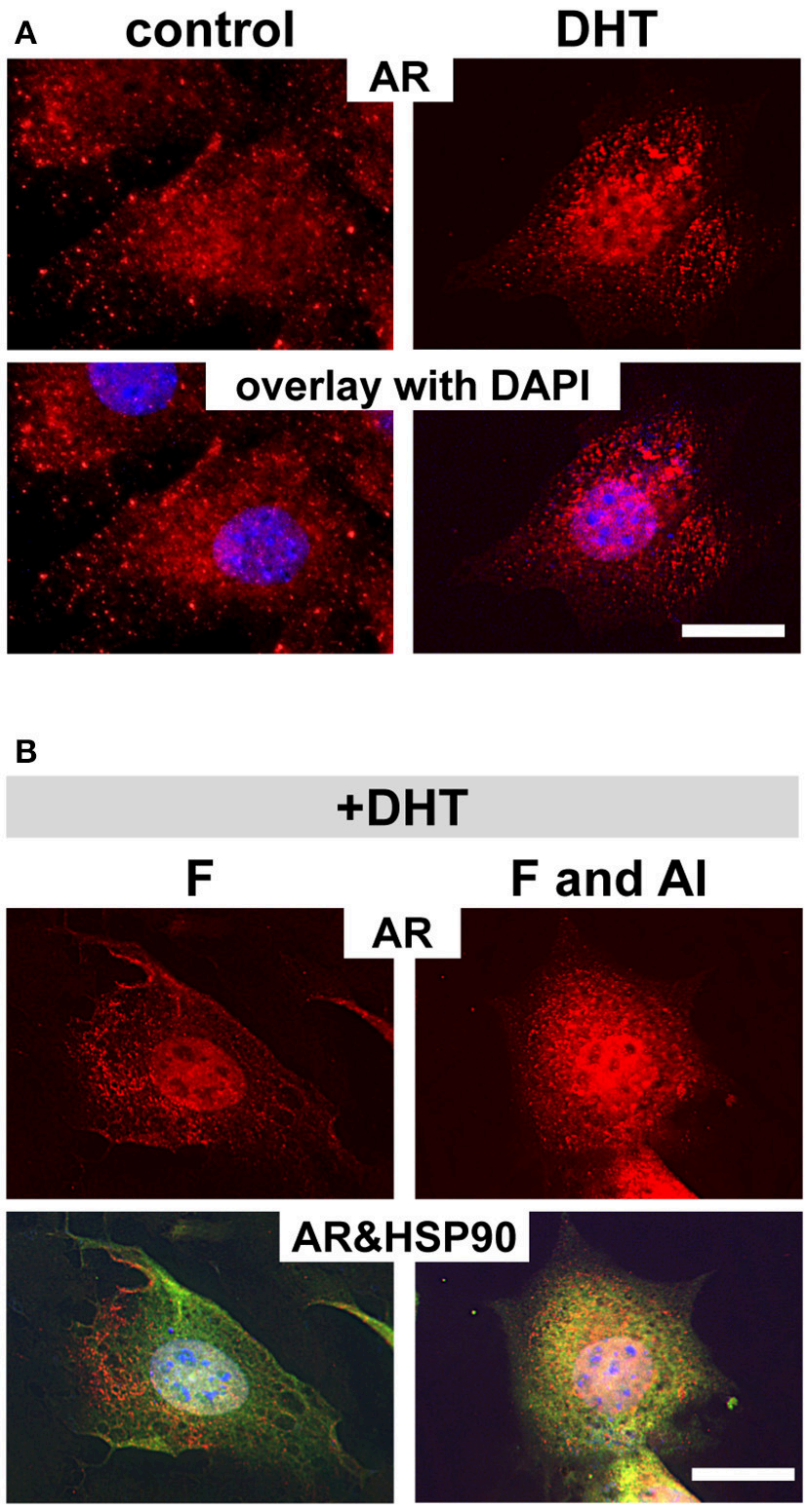
Aluminum is required for fluoride to inhibit AR translocation into nucleus. Immunofluorescent analysis of AR in ALCs cultured in medium containing 2% TCM® serum replacement. **(A)** By adding DHT, increased AR staining (in red) in the nucleus is observed compared with non DHT treated cells (control). **(B)** When cells are treated with DHT, subsequent exposure to 1 mM fluoride does not change the intracellular localization of AR as compared to the control. However, when cells are exposed to 100 μM aluminum, together with fluoride, more AR remains in the cytoplasm. The increased cytoplasmic AR co-localizes with HSP90 (in green), and is seen as the overlaid yellow color. DAPI in blue, Scale bar 25 μm.

Among the nuclear factor family steroid hormone receptors, whose activation strictly depends on the interaction with HSP90, glucocorticoid receptor is the most well-studied steroid hormone receptor (Grad and Picard, 2007). Housley et al. showed that fluoride requires aluminum to stabilize HSP90 binding to glucocorticoid receptor (Housley, 1990). To determine whether aluminum is also required for the fluoride related cytoplasmic stabilization of AR by HSP90 in ameloblasts, we cultured ALCs and examined the intracellular AR localization in presence of fluoride and aluminum. ALCs were grown in serum containing media for 24 h, after which the media was changed to one with an artificial serum substitute (TCM® serum replacement). We found that AR nuclear translocation was stimulated by dihydrotestosterone (DHT) in ALCs (Figure 6A). However, when nuclear translocation was stimulated by DHT, the addition of fluoride alone did not alter the intracellular localization of AR, whereas in the presence of both fluoride and aluminum, more AR remained in the cytoplasm (Figure 6B). *Ar* transcription was not significantly changed in the presence of aluminum and fluoride (data not shown). Further immunostaining showed that the increased cytoplasmic AR in the presence of both fluoride and aluminum co-localized with HSP90 (Figure 6B). An increased AR retention in the cytoplasm was observed in both 10 μM (data not shown) and 100 μM aluminum supplemented culture.

## Discussion

The hypomineralized phenotype of severely fluorosed enamel is due in part to a prolonged retention of enamel matrix proteins (Den Besten, 1986; Wright et al., 1996), likely related to the reduced proteinase activity in fluorosed maturation stage enamel (DenBesten et al., 2002). Free fluoride in the enamel matrix is at micromolar levels (Aoba, 1997), which does not significantly alter the matrix proteinase activity (Tye et al., 2011). Therefore, if KLK4 proteinase activity is reduced in the fluorosed enamel matrix, it should be due to reduced KLK4 synthesis. Supporting this possibility, Suzuki et al. found reduced *Klk4* expression in the enamel organ of rats exposed to 50–100 ppm fluoride in the drinking water (Suzuki et al., 2014). We also confirmed that *Klk4* expression is reduced in maturation stage enamel organs of rats and mice exposed to high levels of fluoride in drinking water, and consistent with reduced *Klk4* expression, we found a significant reduction of KLK4 proteolytic activity in fluorosed maturation enamel matrix.

KLK4 is upregulated during ameloblast maturation, but the mechanism by which KLK4 expression is regulated in ameloblasts has not been investigated. KLK4 expression is known to correlate with prostate and ovarian cancer, and AR signaling in association with TGF-β signaling is well-studied in these tissues/cells. In OV-Mz-6 ovarian cancer cells, expression of KLK4–7 leads to elevated TGFβ-1 signaling (Shahinian et al., 2014). In prostate cancer cells, TGF-β induces AR activation of its target genes (Yang et al., 2014), while AR also represses TGF-β signaling through interaction with Smad3 (Chipuk et al., 2002). Furthermore, in prostate smooth muscle cells, TGF-β1 inhibits AR activation (Gerdes et al., 1998). In ameloblasts, Suzuki and co-workers suggested that TGF-β signaling is a target of fluoride to reduce *Klk4* expression (Suzuki et al., 2014). As there is a cross-talk between AR and TGF-β signaling, and more recent studies report the presence of AR in ameloblasts and that AR activation upregulates *Klk4* expression (Houari et al., 2016; Jedeon et al., 2016), our studies were directed to determine whether fluoride inhibits AR activation in ameloblasts to result in the downregulation of *Klk4* expression.

Both AR and PR are transcription factors for *Klk4*, however, Lai et al. reported that AR interactions with the *Klk4* gene is indirect and requires co-regulators, while PR directly binds to the progesterone response element in the *Klk4* gene (Lai et al., 2009). Activation of AR induces *Klk4* transcription in prostate cancer cells, while activation of PR does so in breast cancer cells (Nelson et al., 1999; Lai et al., 2009). Therefore, *Klk4* transcription seems to be driven by AR and/or PR, depending on the circumstance such as availability of co-regulators and cell types. In ALCs, we found that inhibition of AR signaling by *Ar* silencing or fluoride, resulted in upregulation of *Pgr* and *Klk4* expression. However, in presence of fluoride, nuclear translocation PR was also inhibited resulting in the downregulation of *Klk4* expression, indicating that PR has a dominant transactivation role in *Klk4* transcription, rather than AR in our *in vivo* (with C57BL/6 mice) and *in vitro* (with ALCs) experimental system. Indeed, in our ALC culture, DHT induced nuclear translocation of AR, but did not upregulate *Klk4* expression (data not shown). Jedeon et al. showed that in rat HAT-7 ameloblasts, activation of AR by testosterone (a primary androgen) increased expression of both *Klk4* and *Ar* (Jedeon et al., 2016), also in maturation ameloblasts in castrated rats injected with testosterone, *Klk4* expression was also increased (Jedeon et al., 2016). However, we found that the enamel in testicular feminized (Tfm) mouse, in which AR lacks steroid binding domains (He et al., 1994), was not hypomineralized to the extent that we saw in fluorosed mice (unpublished studies), supporting the possibility AR regulates KLK4 expression to a much less extent than PR in mouse ameloblasts. Clearly, further investigation of AR and PR signaling is necessary to understand their role in tooth development.

We did find reduced immunostaining for TGFB1 and TGFR-2 in fluorosed maturation stage ameloblasts. This finding along with increased expression of Cyclin D1 in fluorosed ameloblasts, suggests that fluoride interacts with a regulatory molecule common to TGF-β, AR, and PR signaling. The 90-kDa heat-shock protein (HSP90) is such a candidate molecule. HSP90 is an abundant molecular chaperone that functions by facilitating protein folding and stabilization (Csermely et al., 1998; Li et al., 2012). The chaperone function of HSP90 to TGF-β receptors (TGFR-1 and TGFR-2) is known to regulate TGF-β signaling activity in a range of normal and oncogenic epithelial cells (Wrighton et al., 2008). Loss of HSP90 function by stabilizing conformation of HSP90, results in ubiquitin-mediated degradation of clients, i.e. TGF-β receptors, and blocks TGF-β-induced Smad2/3 activation and transcription (Wrighton et al., 2008). Also, nuclear factor family steroid hormone receptors including AR and PR are well-known clients for HSP90 (Reddy et al., 2006; Azad et al., 2015). Therefore, an effect of fluoride on HSP90 could reduce TGFR-2 levels, which suppress TGF-β signaling activity, resulting in an downregulated AR transactivation likely via upregulated CCND1 (Knudsen et al., 1999). Simultaneously, nuclear translocation of AR and PR would be suppressed.

The ATPase function of HSP90 N-terminal domain has a critical role in the functioning cycle of HSP90 (Li and Buchner, 2013). ATP binding to HSP90 triggers a conformational change to a closed state, forming a “lid” that closes over the nucleotide binding pocket. When HSP90 reaches a fully closed state, ATP hydrolysis occurs to release ADP and inorganic phosphate, and returns again to the open conformation (Sullivan et al., 2002; Colombo et al., 2008; Zhang et al., 2015). Aluminum fluoride is known to occupy the γ-phosphate-binding site on the nucleotide triphosphate binding proteins, and together with bound nucleoside diphosphate, such as ADP and GDP, it stabilizes the transition state of the proteins (Wittinghofer, 1997). If aluminum fluoride complex occupies the γ-phosphate-binding site in the ATP binding pocket of HSP90, similar to the effects of aluminum fluoride on G-proteins (Li, 2003) and nitrogenase (Renner and Howard, 1996; Schindelin et al., 1997), the ATP binding pocket can accept only ADP, not ATP. HSP90 would then take the closed conformation, but be stabilized in that state, preventing release of the client proteins (such as AR and PR) to inhibit their translocation to the nucleus. Our results showing that fluoride in combination with aluminum inhibited AR nuclear translocation in ALCs in serum free media (i.e. in the use of the artificial serum replacement), provides indirect evidence to support this possibility. Aluminum is a trace element in the normal serum (Wang et al., 1991; Murko et al., 2009; Tomza-Marciniak et al., 2011), and it could explain why we see an effect of fluoride inhibiting AR nuclear translocation in serum containing media, but not in serum free media except with the addition of aluminum.

Finally, it is important to mention that studies of the cellular effects of fluoride require attention to biologically relevant levels of fluoride. We do not yet know how fluoride enters ameloblasts, and the effect of the unique process of enamel mineralization, such as changes in the extracellular pH. *In vivo*, rodents ingest ~10 times the amount of fluoride as humans to obtain similar serum levels and degrees of fluorosis. Humans drinking 3–5 ppm F (1 ppm *F* = 52.6 mM) fluoride supplemented water have a serum fluoride level around 3–5 μM (Guy et al., 1976), similar to the mouse model used in this study which was given water containing 50 ppm F (Zhang et al., 2014). However, *in vitro*, fluoride levels are required to be much higher to show effects similar to those found *in viv*o (Sharma et al., 2008; Li and Buchner, 2013; Lei et al., 2014; Suzuki et al., 2014). We found that ALCs required 1 mM fluoride to result in reduced AR translocation, similar to what was found *in vivo*, and at these fluoride levels, cell proliferation and morphology of the ALCs were not affected, indicating that the effects of fluoride on the ALCs used for these *in vitro* studies are specific and not due to a generalized effect of fluoride on cell toxicity. The need for higher levels of fluoride in ALC culture may be related to the mechanisms by which fluoride may enter the cell, as compared to *in vivo*.

In conclusion, fluoride targets activation of AR and PR and thus inhibits AR- and PR- driven *Klk4* transcription in maturation ameloblasts. Our data support the possibility that this effect occurs through the action of fluoride in combination with aluminum, possibly through an effect on HSP90. These studies raise many questions related to the regulation of ameloblast maturation, and how fluoride can alter this process at a cellular level, to result in fluorosis. The cellular effect of fluoride may be related to fluoride entry into the cell, which may be unique to ameloblasts, and additional studies will be needed to address this possibility. It should also be noted that the *in vitro* studies were all done using female rats and mice. Though there is no convincing evidence that fluorosis is sex-linked, it is reasonable that future studies of fluorosis mechanisms include both male and female mice.

## Author contributions

All authors listed, have made substantial, direct and intellectual contribution to the work, and approved it for publication.

### Conflict of interest statement

The authors declare that the research was conducted in the absence of any commercial or financial relationships that could be construed as a potential conflict of interest.
